# Economics of Contract Practice

**Published:** 1916-04

**Authors:** 


### Economics of Contract Practice

The practical evils of contract practice have been too thoroughly discussed to need reiteration. Yet, viewing the matter broadly, there is no ethical nor economic reason against a contract by an individual, family or society, to secure medical care as needed, for a fixed premium. Every argument that applies to the advisability of fire, life, liability or other insurance, applies to insurance of proper medical care, without pauperism or an overwhelming expense.

The real reasons why Contract Practice has been so violently condemned by the medical profession generally are that, in the first place, it has not usually selected the attendant with due regard to personal choice and natural affiliation of the person or family seeking treatment, nor with due regard to the ordinary properties governing professional competition; and that in the second place, the premium has been inadequate. This latter is the essential weakness of Contract Practice. It is one of the main factors in excluding from competition, a large number of the profession. It requires that services only nominally paid for shall be rendered to those who ought not to seek charity and pauperism is thinly disguised under the veil of a business contract. It tempts to inadequate and unskilled service and to neglect. Many persons entitled to contract attendance realize this fact so well that they do not attempt to avail themselves of the benefits for which they pay.

A study of College Health Activities in the Dec. 1915 issue of the Journal of the Iowa State Medical Society, throws some light on what the proper premium for contract attendance should be. The following annual assessments are made for physical examination, diagnosis of existing abnormal conditions, and general supervision of health but not including treatment of actual sickness nor accident. The fees are supposed to be based on actual expense of maintenance: Princeton $7, Harvard $4, Cornell $6, Stanford $4, Wellesley $5, California $6, Michigan $4, Texas $5, Iowa $4, Kansas $2. When we consider that the age and, in the majority of cases, the sex and social condition of the beneficiaries determine a vulnerability considerably less than the average for all ages and both sexes, and that the expense of actual disease and injury is not included, it is obvious that Contract Practice cannot make a plea for respectful consideration until its premiums are multiplied ten times at least.

